# Biomechanics of Osteoporotic Fracture Fixation

**DOI:** 10.1007/s11914-019-00535-9

**Published:** 2019-11-21

**Authors:** Marianne Hollensteiner, Sabrina Sandriesser, Emily Bliven, Christian von Rüden, Peter Augat

**Affiliations:** 1 Institute for Biomechanics, BG Unfallklinik Murnau, Prof.-Kuentscher-Str. 8, 82418 Murnau am Staffelsee, Germany; 2 Institute for Biomechanics, Paracelsus Medical University, Salzburg, Austria; 3 Department of Trauma Surgery, BG Klinikum Murnau, Murnau, Germany

**Keywords:** Bone, Fracture, Osteoporosis, Biomechanics, Osteosynthesis, Fragility fracture

## Abstract

**Purpose of Review:**

Fractures of osteoporotic bone in elderly individuals need special attention. This manuscript reviews the current strategies to provide sufficient fracture fixation stability with a particular focus on fractures that frequently occur in elderly individuals with osteoporosis and require full load-bearing capacity, i.e., pelvis, hip, ankle, and peri-implant fractures.

**Recent Findings:**

Elderly individuals benefit immensely from immediate mobilization after fracture and thus require stable fracture fixation that allows immediate post-operative weight-bearing. However, osteoporotic bone has decreased holding capacity for metallic implants and is thus associated with a considerable fracture fixation failure rate both short term and long term. Modern implant technologies with dedicated modifications provide sufficient mechanical stability to allow immediate weight-bearing for elderly individuals. Depending on fracture location and fracture severity, various options are available to reinforce or augment standard fracture fixation systems.

**Summary:**

Correct application of the basic principles of fracture fixation and the use of modern implant technologies enables mechanically stable fracture fixation that allows early weight-bearing and results in timely fracture healing even in patients with osteoporosis.

## Introduction

The healing of a fractured bone requires immobilization by conservative measures (i.e., cast or orthosis) or by surgical fixation with osteosynthesis implants (i.e., screws, plates, or nails). Fractures of osteoporotic bones are particularly challenging to treat for several reasons. First, the nature of osteoporotic bone itself, because of its decreased density and its increased brittleness, tends to fracture into more and smaller individual fragments creating more complex fractures than healthy bone [1]. This requires considerable surgical skills to achieve reduction and efficient implants for stable retention of the fracture. Second, osteoporotic fractures occur in elderly people who have decreased capacity to manage functional limitations [2]. Age-associated reductions in sense of balance, coordination, and proprioception combined with reduced vision result in uncoordinated limb loading and increased risk of falling [3]. Also, prescribed limitations in weight-bearing after fracture fixation often cannot be complied with, and lead to overloading of the fracture fixation constructs. This requires the fracture fixation to provide maximal stability in order to withstand immediate full weight-bearing. Third, osteoporosis is typically not the only condition elderly individuals have to deal with. Increasing age substantially increases the prevalence of comorbidities and the decline in organ function (heart, lung, kidneys, and liver). This makes elderly individuals much more vulnerable to post-traumatic complications and necessitates quick and minimally invasive surgery as well as rapid mobilization [4]. In terms of fracture fixation, this again requires sufficient mechanical stability to immediately mobilize the patient. Finally, osteoporotic bone has deteriorated mechanical properties reflected in porous cancellous and thin cortical bone resulting in reduced resistance to loading by rigid osteosynthesis implants. Consequently, implant loosening, implant cut out, and peri-implant fractures are frequent complications of osteoporotic fracture treatment [5]. Osteosynthesis implants thus need to either be designed to withstand loosening or be otherwise reinforced or augmented to prevent this type of failure. Overall, fracture fixation in elderly individuals with osteoporosis requires enduring stable fracture fixation with unrestricted load-bearing capacity. In this manuscript, the current strategies to provide sufficient fracture fixation stability will be reviewed with a particular focus on fractures that frequently occur in elderly individuals with osteoporosis [6] and which require full load-bearing capacity, i.e., pelvis, hip, ankle, and peri-implant fractures.

## Hip Fractures

Fractures of the proximal femur constitute a huge health and economic burden for societies all over the world [7] with incidence rates of up to 400 fractures per 100,000 individuals per year [8] and considerably higher fracture risk in women [9]. Thirty percent of hip fractures are caused by low-energy trauma and another 69% are spontaneous fractures [10]. Individuals suffering fragility fractures experience a drastic increase in mortality and morbidity. Excess mortality rates can be as high as 36% within the first year after hip fracture [11]. Their level of mobility and their quality of life is decreased and they often need care and supervision [12]. Only approximately one third of patients regain their previous mobility after a hip fracture [13].

Although cortical bone in the proximal femur is mainly responsible for the whole bone strength, cancellous bone still contributes to about 10% to the total strength in stance [14, 15] and 35% during a sideways fall [15]. Trauma mechanisms of femoral neck fractures may either be direct, e.g., fall onto the greater trochanter or a forced external rotation of the leg, or indirect, if muscle forces overwhelm the internal strength of the femur. As the femoral neck is intracapsular and thus not covered by periosteum, periosteal bone apposition is unable to compensate for cortical thinning caused by endosteal resorption. Thus, due to cortical thinning and trabecular bone loss, the femoral neck in particular loses strength and becomes susceptible to fracture. Similarly, as the lateral cortex of the trochanter becomes thinner during aging, it has a higher potential to buckle during a fall impacting the hip [16].

The AO/OTA fracture classification (Table 1) distinguishes extra-articular fractures in the trochanteric area (31-A), intra-articular fractures in the neck area (31-B), and fractures of the femoral head (31-C) [17].

**Table 1:**
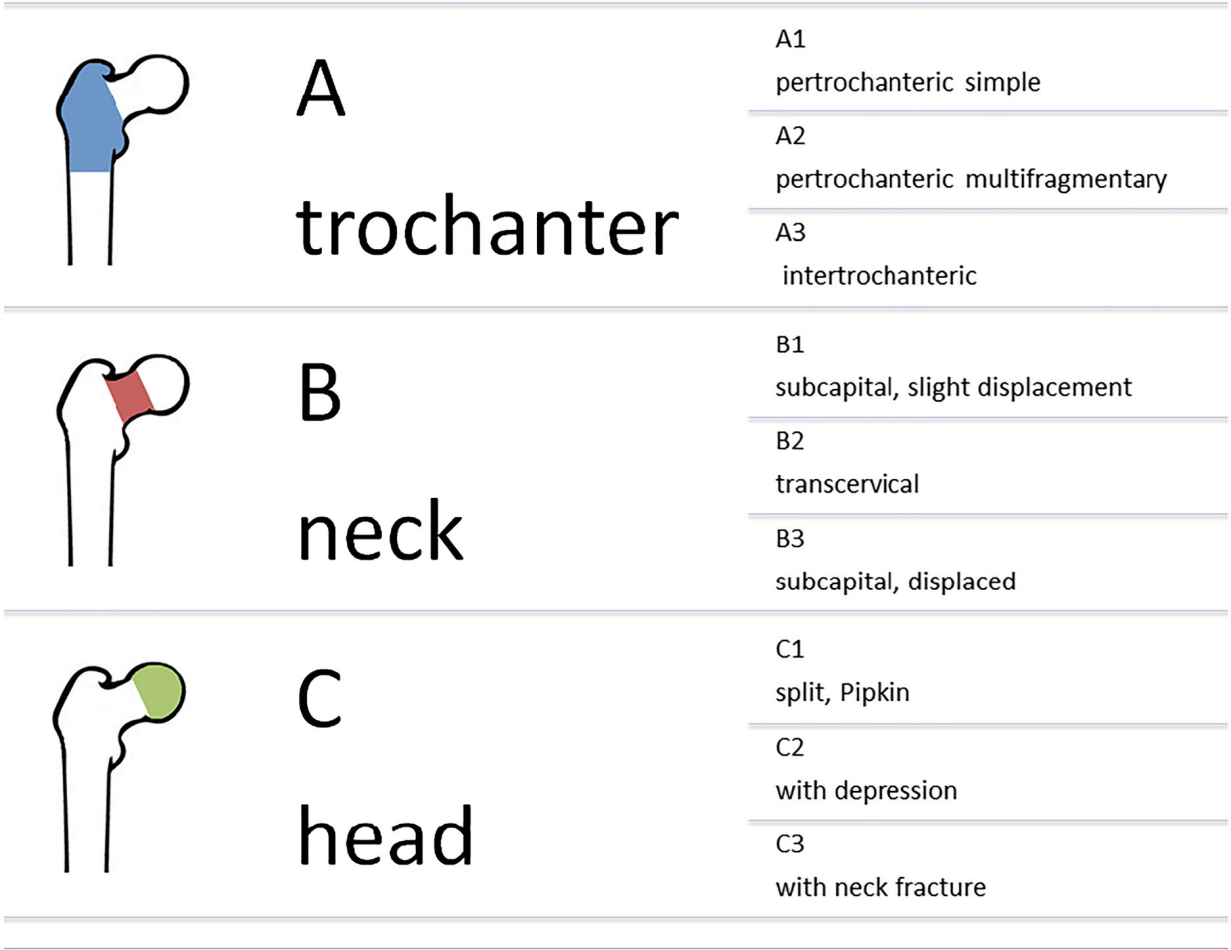
The AO classification of proximal femur fractures (A: trochanteric area, B: the neck area, C: head area)

The overall incidence rates of trochanteric and cervical fractures are similar, but the injuries possess etiologic and demographic differences. Women with trochanteric fractures are older, have more severe and generalized bone loss, and more frequently suffer from other osteoporotic fractures [18]. Fractures of the trochanter are most frequently multifragmentary pertrochanteric (A2), simple 2-fragment fractures (A1) in one third of the cases and rarely occur as reverse fractures (A3) [10]. Cervical fractures are frequently classified according to Pauwels considering the inclination of the fracture line or according to Garden considering their prognosis and potential complications [19–21]. The more recent classification from the AO, which partly incorporates the Pauwels classification, additionally includes the fracture level and degree of displacement. Femoral neck fractures are thus classified as subcapital (31-B1), transcervical (31-B2), or basicervical (31-B3) fractures. Furthermore, fractures can be distinguished by being described as impacted, displaced, non-displaced, simple, multifragmentary, or shear [17]. Femoral neck fractures are often simply described as displaced or non-displaced because intra- and interobserver reliability is poor when using the various fracture classifications [20]. A displaced fracture is characterized by any detectable displacement of the fracture while non-displaced or impacted fractures show either an impacted valgus or simply no visible displacement [9].

## Treatment of Trochanteric Fractures

While there is a general consensus on treatment of stable fractures (A1, A2), the best way to treat unstable fractures remains controversial [17]. Simple trochanteric fractures are treated by extra- or intramedullary devices [16, 22], mostly sliding hip screws and cephalo-medullary nails. While it appears that the use of cephalo-medullary nails is becoming more and more popular, there is no clear clinical evidence on the superiority of any surgical treatment method yet available [23]. For highly unstable subtrochanteric and reverse obliquity fractures, long cephalo-medullary devices have been shown to be the most successful treatment option [12].

Due to the lack of clear clinical evidence regarding the optimal surgical treatment, implant choice is often based on biomechanical performance. Biomechanical studies consistently show that cephalo-medullary nails perform biomechanically better than sliding hip screws, as they combine the advantages of the two options through a controlled impaction of the fracture and a closer-to-central weight-bearing axis in the femoral shaft [24]. Concerning very unstable fractures, novel nail designs with interlocking lag screws provide improved mechanical performance over nails with single lag screws [25]. For extremely unstable fracture situations, cerclage wiring or auxiliary plates have shown to improve biomechanical stability [26]. It appears that these biomechanical findings translate well into clinical practice [26, 27].

Newly emerging implants such as angle-stable locking plates allow biological flexible fracture fixation based on the principle of an internal fixator [28]. Although they show excellent biomechanical performance [29], their clinical results are rather discouraging with catastrophic rates of nonunion (19%) and mechanical failure (38%) [30]. This contradiction might be explained by the bone healing mechanism in trochanteric fractures which differs from the healing mechanism in diaphyseal fractures. Diaphyseal fractures benefit from motion at the fracture site which is promoted by locking plates while trochanteric fractures heal more like cancellous bone fractures which benefit from accurate reposition and bone compression. Thus, despite biomechanical advantages, the locked plate may not induce the adequate healing response [31] and the plate might experience overloading during cyclic loading peak stresses, crack initiation, and propagation [32]. Thus, as long as the clinical evidence is still lacking, cephalo-medullary devices seem to be preferable over extramedullary implants when early weight-bearing is indicated.

## Treatment of Cervical Fractures

Treatment of femoral neck fractures in elderly should enable early mobilization [9] and provide sufficient mechanical stability until the fracture has healed. It has been shown that the healing process heavily relies on the mechanical stability of the bone-osteosynthesis construct, especially in comminuted fractures [33•]. Internal fixation by osteosynthesis is considered to be the standard form of treatment for non-displaced femoral neck fractures [11, 12]. Non-comminuted, stable fractures are able to be securely fixated with use of only cannulated or hip screws [33•]. Commonly available techniques for unstable fractures include implementation of cannulated screws, hip screw systems (also with additional anti-rotation screws), proximal femur plates, and cephalo-medullary nails.

The main advantage of cannulated screws is that they are less invasive [9], apply compression to the fracture gap, and allow sliding of the head along the shaft axis, thus accelerating healing [33•]. The main problem when placing screws in osteoporotic femurs is that the central area of the femoral neck often lacks cancellous bone. Thus, biomechanical studies mainly focus on the necessary number, diameter, and position of screws, as such factors are of critical importance to resist displacement of the fracture gap and achieve bony union. Screw stability relies on anchorage at the lateral cortex on one side and the subchondral bone of the femoral head on the other [34]. Biomechanical studies report that screws with cortical support lead to higher stability than screws anchored in the cancellous bone alone [35]. Furthermore, fracture stability is increased when 3 instead of only 2 screws are placed [36]. Although additional screws increase construct stability, they may weaken the lateral cortex, necessary for proper screw anchorage [37]. From a biomechanical point of view, the recommended screw construct for femoral neck fractures is an inverted triangle configuration with three parallel screws. The parallel screw configuration enables sliding of the head fragment along the neck axis and thus further impaction of the neck [38]. The use of washers is additionally recommended, especially for osteoporotic bone, as they generate more compression in the fracture gap and prevent screw heads from penetrating the lateral cortex [39].

More vertically oriented fractures should be stabilized with sliding hip screws [40], which have been shown to be biomechanically superior to cannulated screws [41]. The potential risk of rotational malalignment is reduced by using additional anti-rotation screws [42]. A recently introduced novel hybrid between cannulated and sliding hip screws was reported to provide both rotational stability and controlled collapse of the femoral neck, combining the advantages of the individual options through smaller diameter sliding screws in a lateral locking plate. The novel implant has already shown reduced nonunion rates; however, more clinical evidence of its potential benefits is needed [43].

Another issue when treating femoral neck fractures is the shortening of the femoral neck. Historically this concern was accepted as a standard clinical outcome, but studies report severe impacts on patient’s physical function [44]. In order to address this problem, new length-stable implants such as fully threaded cancellous screws, divergent cancellous screws, or proximal femoral locking plates have been introduced [9] with conflicting clinical results. A novel internal fixation technique combines two fully threaded divergent screws placed in the head and neck in combination with a sliding hip screw or dynamic helical blade, achieving a non-sliding construct. In 94% of patients treated with this method, the fracture healed with minimal shortening at the fracture site [45]. Catastrophic failure rates of 37% were reported in novel length-stable locking plates, leading authors to recommend against the usage of such an implant for the treatment of femoral neck fractures [46].

Despite all these developments, the rate of fixation failure with need for reoperation after femoral neck fracture osteosynthesis remains at around 40% [47]. Age and low BMD have been identified as the most significant covariates for failure [48]. Thus, increased patient age and osteoporosis lead to a more frequent use of hip arthroplasty and has indeed shown to reduce reoperation rates and result in better functional outcome scores compared to osteosynthesis [11]. Hip arthroplasty as a technique provides a wide variety of options, including hemi or total hip replacement, fixed- or modular neck deigns, cemented or uncemented stems, and uni-, bi-, or even tripolar heads [9]. The decision to implant either total or hemiarthroplasty should be guided by several factors including the patient’s age, activity level, and remaining life span.

## Ankle Fractures

**Fig. 1:**
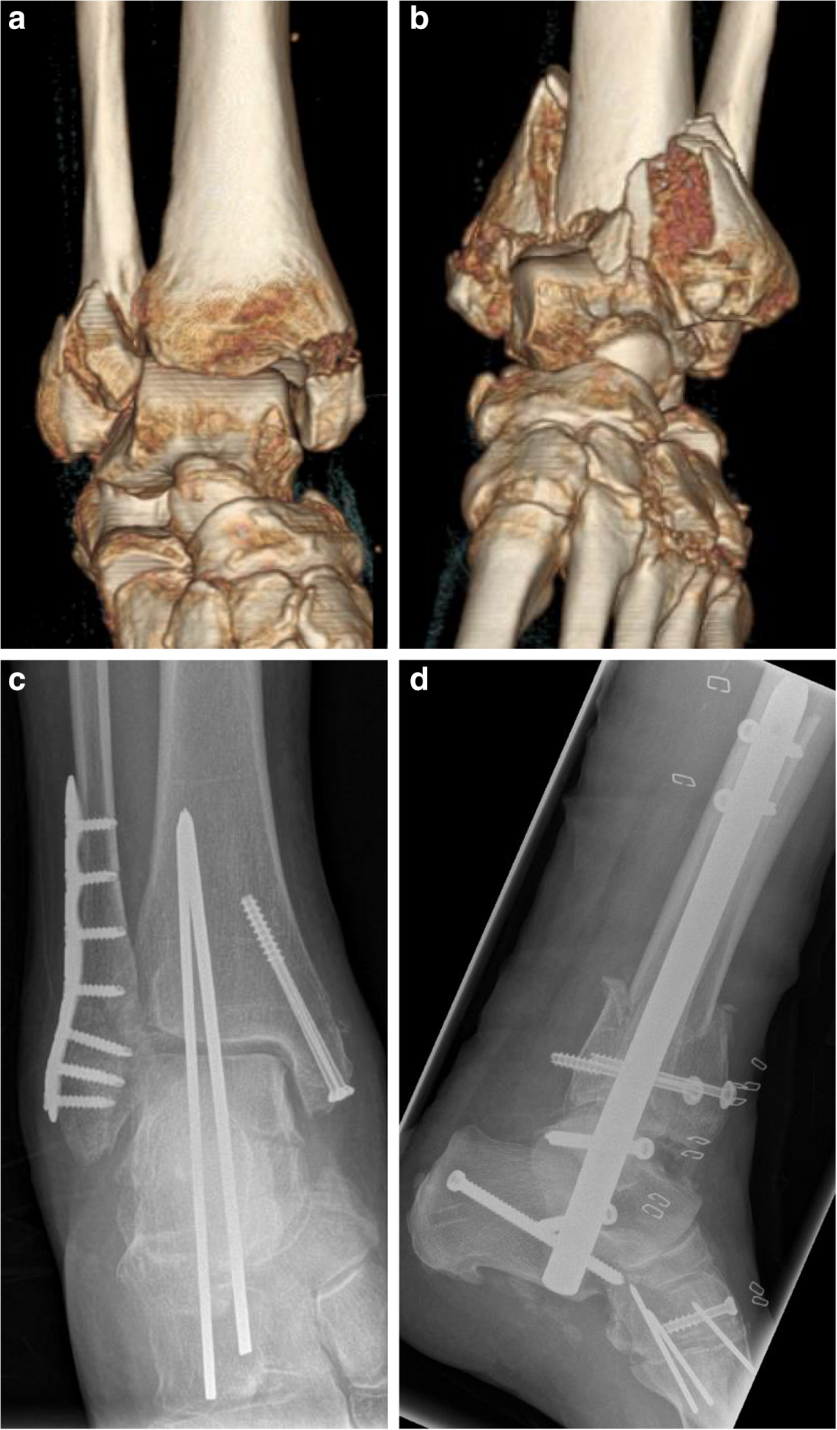
77-year-old female with bilateral complex displaced ankle fractures (**a**, **b**) and an associated Lisfranc injury in the left foot (**b**). Due to vulnerable soft tissue conditions, the right ankle was fixed using open reduction and internal angle-stable plate osteosynthesis of the distal fibula, temporary ankle joint Kirschner wire fixation, and lag screw fixation of the medial malleolus (**c**). The complex fracture on the left required primary transarticular tibiotalocalcaneal nailing (**d**)

With increasing life expectancy, there is an increasing incidence of unstable ankle fractures, which affect mostly women and individuals with poor bone quality. The predominant trauma mechanism in 61% of fracture cases is a fall from standing height [49]. Age and osteoporosis are both considered to be risk factors for ankle fractures. When combined with comorbidities such as diabetes and obesity, the post-operative risk of nonunion is increased and makes fracture treatment challenging [50–52•,^53^,^54^]. Ankle fractures are classified according to Danis-Weber type A, B, or C for the lateral malleolus [55, 56] and AO 43 for the distal tibia and fibula [57]. Non-operative treatment with a cast is only considered to be an option in cases of stable and non-displaced fractures, including isolated medial malleolar or isolated fibular fractures without syndesmotic rupture or instability [58–60]. Open reduction and internal fixation is required in all other more severe cases, in order to obtain a mechanically stable situation and restore the joint’s functionality. For elderly patients who require early mobilization, the fracture fixation needs to be stable enough to allow immediate weight-bearing [61]. Depending on the type and severity of the fracture, stability can be achieved by internal fixation with plate osteosynthesis, intramedullary nailing, lag screws, or a combination thereof (Fig. 1).

Medial malleolar fractures can occur either as an isolated fracture or in combination with lateral malleolar and pilon fractures. If surgical intervention is required, this fracture can be reduced and stabilized by a lag screw, buttress plating, or wiring techniques [62, 63]. Whereas malleolar fractures are mainly caused by rotational trauma, tibial plafond fractures, also known as pilon fractures, occur most often due to impaction of the articular surface [64, 65]. The fragments should primarily be reduced by an external fixator until sufficient reduction of soft tissue swelling is achieved. In a secondary surgical intervention, the focus is on anatomical reduction of the articular surface, which should be performed in the posterior to anterior direction [65, 66]. In a recent study, it was shown that anterolateral plating may not be sufficient in stabilizing the medial malleolar fragment [67]. To achieve sufficient stability, supplementation with an additional medial plate is recommended [65, 68]. Regarding metaphyseal fragments, in rather simple fractures, absolute stability can be reached by the use of lag screws, whereas in comminuted fracture patterns, bridging constructs provide adequate stability [65]. Additional stability can be achieved by using the tibia for additional screw anchorage through trans-syndesmotic screw fixation even with an intact syndesmosis [60]. Depending on the fracture classification, such tibiofibular syndesmotic fixation is also recommended for Weber type B (fracture at the level of the syndesmosis) and type C (fracture proximal to the syndesmosis) fractures with partially torn or disrupted syndesmosis, in order to restore physiological tension and avoid talar displacement and post-traumatic osteoarthritis [69, 70].

Distal fibula fractures are commonly stabilized by either interfragmentary screws and neutralization plates or by plating alone. The controversy of whether locking plates are required for the fixation of lateral malleolar fractures has been recently investigated in a meta-analysis of biomechanical studies [71]. It has been found that both locking as well as non-locking plate constructs provide sufficient mechanical stability for the fixation of fibula fractures. Locking plates may show mechanical benefits compared to conventional plates for the fixation of fractures in highly osteoporotic bone. Intramedullary fixation of the distal fibula, instead of plating, may reduce soft tissue trauma due to incisions being smaller, as wound healing and infections play a crucial role in the clinical outcome of geriatric patients [72–74].

In very poor bone quality, internal fixation constructs can be augmented with cement like polymethylmethacrylate (PMMA) or resorbable calcium phosphate [75]. Both augmentation options increase construct stability and pull-out strength [60], as well as enable early weight-bearing and lower the risk of post-operative implant failure [76]. Tibiotalocalcaneal fusion is only considered as a last resort for pain reduction, and achieves this by eliminating joint motion. This complete stiffening of the ankle and hindfoot remains as an option for patients with minimal to no possibility of mobilization, or if other internal fixation techniques have been unable to provide sufficient stability [60, 77].

In general, the management of ankle fractures in elderly people with osteoporosis needs to be individually considered and the decision-making process can be based on functional demands and the presence of comorbidities with a primary aim of functional restoration allowing early post-operative mobilization [61].

## Pelvis Fractures

The prevalence of pelvis fractures caused by low-energy trauma, such as falls from standing height, has drastically increased. Most pelvis fractures are linked to osteoporosis and occur in individuals that are 60 years and older [78]. The number of low-energy, fall-related fractures outnumbers high-energy fractures by 9 to 1 [6] and is expected to substantially increase [79]. Pelvis fractures constitute a significant clinical problem [6] [80] as they cause intense pain and immobility, impair the quality of life, and lead to loss of patients’ independence [81]. In the elderly population, pelvis fractures are associated with a high morbidity and mortality with up to 27% of patients dying within 1 year of surgery [82, 83].

**Fig. 2:**
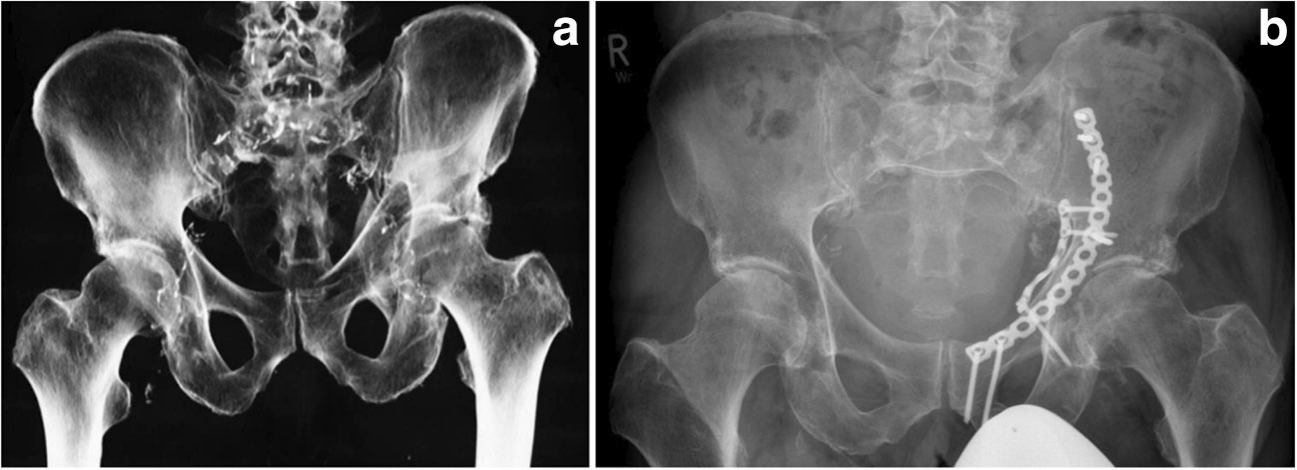
78-year-old male following fall from an apple tree resulting in a comminuted acetabular fracture with displaced quadrilateral plate and acetabular dome also involving the anterior column (**a**). The fracture was fixated using a small fragment plate and cortical screws. An additional medial infrapectineal small fragment plate was placed to stabilize the displaced quadrilateral plate (**b**)

The pelvis can be thought of as a ring-shaped structure with anterior and posterior components. The biomechanically relevant and load-bearing structures are located in the posterior area of the pelvis. Fractures in this area lead to a biomechanical weakening of the load-bearing portion of the ring system and require surgical stabilization. Isolated fractures in the anterior region, where symphysis and adjacent pubic and ischial sections are located, do not lead to substantial mechanical weakening of the ring as these sections do not bear considerable loads but merely function as “bumpers” during walking. Osteoporosis-induced bone degeneration affects the posterior region of the pelvic ring especially, at the triangular surfaces on either side of the sacral base and the alae of the sacrum. Thus, typical fall-induced fracture patterns in the elderly include bilateral fractures in the sacral ala and iliac wings and compression of the lateral pelvis into the superior pubic ramus resulting in pelvic ring collapse and larger fracture displacements [84]. Recently, it has been reported that with increasing activity levels of elderly patients, there has been a shift towards more severe injury patterns [78] (Fig. 2).

The OA/OTA classification distinguishes between stable (A), rotationally unstable (B), and rotationally and vertically unstable (C) fractures. While “type A” fractures are rarely treated surgically, “C” fractures require surgical fixation due to loss of biomechanical stability. “Type B” fractures may require surgical treatment, in particular if immobilizing pain persists, fractures dislocate, or do not heal [85]. In order to address the specific situation of fractures in elderly, a dedicated classification system for fragility fractures of the pelvis (FFP) has been developed [81]. The FFP focuses on the degree of instability which poses the main basis for deciding whether to operate on the fracture or not.

The main objective of pelvic fracture treatment is pain relief and rapid mobilization with early full weight-bearing, as deemed appropriate regarding the patient’s level of pain [83]. There is a need for specific surgical concepts for pelvic ring fractures in the elderly due to different fracture morphologies. Less invasive techniques including splinting and bridging in closed reduction are preferred over open reduction and internal fixation if sufficient mechanical stability can be achieved. Several biomechanical studies investigated construct stiffness and fracture displacement as measures of mechanical stability [81]. A widely accepted surgical technique is the use of percutaneous trans-sacroiliac screws inserted into the first and/or second sacral vertebral bodies [86]. Placement of two iliosacral screws achieves higher biomechanical stability than just one, and, similarly, longer screws achieve a higher stability than shorter ones [86, 87]. Clinically, screws often fail by loosening or unscrewing [85] due to reduced bone quality [81] which can be effectively addressed by using cannulated screws and bone cement to augment the fixation site [88, 89]. Bone cement is also an option for isolated and incomplete compression fractures [83] in which the cement is injected via a needle accessing the sacrum via the sacrum ala. This sacroplasty technique has been effective in decreasing pain and improving quality of life also due to the analgesic effect of bone cement [90].

Fractures of the ilium lateral to the sacroiliac joint require bridging constructs like plate osteosynthesis [83] which have shown to be biomechanically superior when compared to single retrograde screw fixation [91]. Another possibility is transsacral bar osteosynthesis, where a threaded bar is inserted through the sacral corridor of the first sacral vertebra. The advantage of this bridging technique is that washers used to fix the bar prevent the screw heads from penetrating the bone. Therefore, construct stability does not depend on the trabecular bone affected by osteoporosis but rather on the cortical bone of the posterior ilium [92]. Unstable posterior ring fractures are treated with lumbopelvic fixation by means of sacroiliac screw osteosynthesis to achieve both horizontal and vertical stability [83]. Screws are therefore placed into the pedicle of the fifth lumbar vertebra and into the posterior ilium and then interconnected with a vertical rod. This so-called triangular osteosynthesis is more biomechanically stable than iliac screw osteosynthesis [93]. Posterior ring lesions are frequently combined with lesions of the anterior pelvic ring [81]. These injuries require surgical stabilization of both rings [94] as the fixation of only one side of the pelvis will not close the pelvic ring, subsequently leading to instability, higher stress in the placed osteosynthesis, and early implant failure. Additionally, the healing process will likely be decelerated.

External fixation of pelvic ring fractures is often associated with complications like pin track infections, pin loosening [81, 95], and impairment of nursing, bathing, and early mobilization [95]. This minimally invasive fixation principle can also be employed subcutaneously by combining pedicle screws with curved rods placed over the pelvis [81]. Due to placement of rod closer to the bone surface, the subcutaneous placement has mechanical advantages compared to standard external fixator configurations [96].

## Periprosthetic Fractures

The surgical treatment of osteoporotic fractures with metallic implants provides the opportunity for stable fracture fixation and the benefit of early weight-bearing for the patient. However, due to the vast differences in elasticity between the metallic implant and the osteoporotic bone, this method carries a substantial risk for fractures of the bone adjacent to the metal implants, so-called peri-implant or periprosthetic fractures. Although peri-implant fractures occur in essentially all long bone fracture fixations, they are by far the most frequently associated with fracture fixations of the femur.

**Fig. 3:**
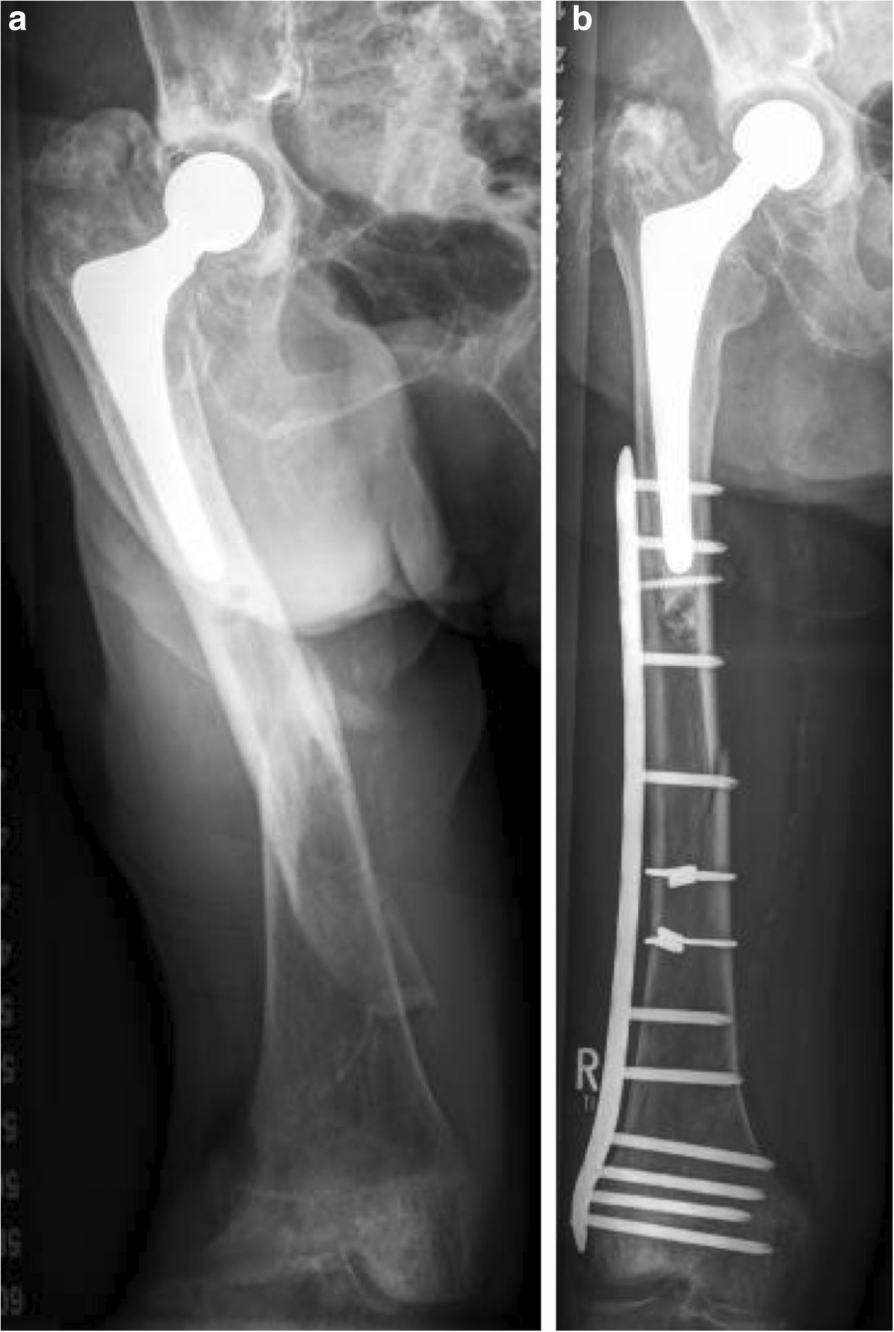
Displaced periprosthetic femur fracture below the tip of the cemented arthroplasty stem (Vancouver type C) in a 90-year-old female treated by open reduction and internal fixation using two cerclages and a locking plate system

Periprosthetic fractures at the femur (PFFs) are commonly described using the Vancouver classification. According to their region of fracture occurrence, PFFs are divided into trochanteric fractures (type A), stem level fractures (type B), and fractures distal to the stem (type C, Fig. 3) [97]. The classification of type B fractures depends on the status of the prosthesis: in type B1, the prosthesis is well-fixed, whereas in type B2, the prosthesis is loose, and in type B3, the prosthesis is loose with the addition of poor bone stock [98]. Type B fractures are reported to make up approximately 75% of all cases [99, 100].

The treatment of PFFs is demanding due to a combination of many factors: a fractured, often osteoporotic bone supporting a total hip prosthesis, which can also sometimes be cemented to anchor the prosthesis in poor bone stock. This scenario usually requires open reduction and internal fixation and eventually revision of the hip prosthesis. Displaced trochanter fractures (PFF type A) are challenging to treat as the trochanter fragment experiences shear and tension during weight-bearing. Thus, combinations of grip plates and cable or cerclage wires have been shown to be biomechanically advantageous [101] and are commonly applied clinically despite considerable complication rates [102]. Type B1 fractures should be treated by reduction and fixation with an invasive plate osteosynthesis, while B2 fractures require revision surgery with the implantation of a longer hip stem. B3 fractures require “salvage” procedures like a megaprosthesis or supplemental fixation with an allograft. Type C fractures, which occur below the hip stem, can be treated by fixation with long plates or by a combination of cerclages and struts [100].

A challenging factor in the treatment of type B PFFs is the presence of the prosthetic hip stem, which is quite often cemented. While sufficient fixation of the fracture should be achieved, the integrity of the cement mantle must be maintained and the stem should not be damaged by screws to avoid stem loosening or breakage [103•]. Solutions have been achieved by tangentially placing screws around the hip stem by the use of locking attachment plates, which are compatible with conventional locking compression plates [104], hook plates [105], or locking plates [106]. Modern locking plates allow polyaxial as well as bicortical screw placement around a hip stem [106] and sustain up to 40% higher failure loads than constructs of locking plates with additional superiorly mounted attachment plates [107]. Due to the voids of cancellous bone in the osteoporotic femur, the bicortical anchorage of screws in the cortex is essential to achieve biomechanical stability [106]. Bicortical tangential screw placement still carries the risk of the breach of cement mantle integrity and may lead to an early loosening of a cemented hip stem [108] due to cement mantle damage and crack formation. As the risk of a crack decreases with the distance of the screw in relation to the stem screws should be placed outside or at the periphery of the cement [109].

In comminuted fractures, one plate may not be stable enough for early weight-bearing, thus requiring stiffer and longer plates, additional cerclage wiring [110], or even double-plating constructs to prevent osteosynthesis failure [107]. These constructs require a large surgical approach and seriously deteriorate the healing capacity, thus serving as a permanent mechanical stabilizer. They may be a viable rescue option, especially for patients with unstable comminuted fractures, severe osteoporosis, over-weight, or other noncompliant patients with a need for immediate post-operative full weight-bearing [111]. In unstable type B3 and type C fractures, a long stem revision in combination with a long plate often provides the only option for treatment [112, 113].

## Conclusion

Fracture fixation in elderly individuals with osteoporosis requires stable fracture fixation that enables early mobilization of the patient and prevents fixation failure due to loosening, cut out, or peri-implant fracture. The consequent application of existing osteosynthesis techniques and choice of the most stable implant configuration can guarantee adequate fixation stability. Certain fracture situations may benefit from augmentation by cementing techniques or employing additional hardware such as auxiliary plates, cerclage wires, or double-plating techniques. Novel implant techniques that have shown biomechanical benefits for osteoporotic fracture fixation still need to be evaluated with respect to their clinical performance.
